# It is not just menopause: symptom clustering in the Study of Women’s Health Across the Nation

**DOI:** 10.1186/s40695-017-0021-y

**Published:** 2017-07-27

**Authors:** Siobán D. Harlow, Carrie Karvonen-Gutierrez, Michael R. Elliott, Irina Bondarenko, Nancy E. Avis, Joyce T. Bromberger, Maria Mori Brooks, Janis M. Miller, Barbara D. Reed

**Affiliations:** 1 Department of Epidemiology, School of Public Health, University of Michigan, 1415 Washington Heights, Suite 6610 SPH I, Ann Arbor, MI 48109-2029 USA; 2 Department of Biostatistics, School of Public Health, University of Michigan, Ann Arbor, USA; 3 Department of Social Sciences and Health Policy, Wake Forest School of Medicine, Winston-Salem, USA; 4 Departments of Epidemiology and Psychiatry, University of Pittsburgh, Pittsburgh, USA; 5 Department of Epidemiology, University of Pittsburgh, Pittsburgh, USA; 6 School of Nursing, University of Michigan, Ann Arbor, USA; 7 Department of Family Medicine, University of Michigan School of Medicine, Ann Arbor, USA

**Keywords:** Symptom clusters, Sleep, Pain, Fatigue, Vasomotor symptoms, Psychological symptoms, Menopause, Aging, Latent transition analysis

## Abstract

**Background:**

Patterns of symptom clustering in midlife women may suggest common underlying mechanisms or may identify women at risk of adverse health outcomes or, conversely, likely to experience healthy aging. This paper assesses symptom clustering in the Study of Women’s Health Across the Nation (SWAN) longitudinally by stage of reproductive aging and estimates the probability of women experiencing specific symptom clusters. We also evaluate factors that influence the likelihood of specific symptom clusters and assess whether symptom clustering is associated with women’s self-reported health status.

**Methods:**

This analysis includes 3289 participants in the multiethnic SWAN cohort who provided information on 58 symptoms reflecting a broad range of physical, psychological and menopausal symptoms at baseline and 7 follow-up visits over 16 years. We conducted latent transition analyses to assess symptom clustering and to model symptomatology across the menopausal transition (pre, early peri-, late peri- and post-menopausal). Joint multinomial logistic regression models were used to identify demographic characteristics associated with premenopausal latent class membership. A partial proportional odds regression model was used to assess the association between latent class membership and self-reported health status.

**Results:**

We identified six latent classes that ranged from highly symptomatic (LC1) across most measured symptoms, to moderately symptomatic across most measured symptoms (LC2), to moderately symptomatic for a subset of symptoms (vasomotor symptoms, pain, fatigue, sleep disturbances and physical health symptoms) (LC3 and LC5) with one class (LC3) including interference in life activities because of physical health symptoms, to numerous milder symptoms, dominated by fatigue and psychological symptoms (LC4), to relatively asymptomatic (LC6). In pre-menopause, 10% of women were classified in LC1, 16% in LC2, 14% in LC3 and LC4, 26% in LC5, and 20% in LC6. Intensity of vasomotor and urogenital symptoms as well as sexual desire) differed minimally by latent class. Classification into the two most symptomatic classes was strongly associated with financial strain, White race/ethnicity, obesity and smoking status. Over time, women were most likely to remain within the same latent class as they transitioned through menopause stages (range 39–76%), although some women worsened or improved. The probability of moving between classes did not differ substantially by menopausal stage. Women in the highly symptomatic classes more frequently rated their health as fair to poor compared to women in the least symptomatic class.

**Conclusion:**

Clear patterns of symptom clustering were present early in midlife, tended to be stable over time, and were strongly associated with self-perceived health. Notably, vasomotor symptoms tended to cluster with sleep disturbances and fatigue, were present in each of the moderate to highly symptomatic classes, but were not a defining characteristic of the symptom clusters. Clustering of midlife women by symptoms may suggest common underlying mechanisms amenable to interventions. Given that one-quarter of midlife women were highly or moderately symptomatic across all domains in the pre-menopause, addressing symptom burden in early midlife is likely critical to ameliorating risk in the most vulnerable populations.

**Electronic supplementary material:**

The online version of this article (doi:10.1186/s40695-017-0021-y) contains supplementary material, which is available to authorized users.

## Background

Studies of the menopausal transition have found that women who experience hot flashes are at increased risk of experiencing additional symptoms, such as anxiety, depression or sleep, both concurrently and longitudinally [1–4]. Woods [5] proposed that symptom clustering may suggest common underlying mechanisms, and may identify women at risk of adverse health outcomes or, conversely, more likely to experience healthy aging. Studies of breast cancer patients have reported clustering of depression, fatigue and sleep over time [6] and of fatigue, pain and psychological symptoms [7] Studies of cardiovascular disease suggest that symptom clustering differs by age, with younger patients reporting more symptoms and older patients reporting fewer but more diffuse symptoms [8]. Despite interest in menopausal symptoms, relatively few studies have evaluated symptom clustering among midlife in women [5, 9–17].

Research conducted in nonclinical populations of midlife women find that women report different symptom patterns, with clusters generally based on symptom intensity and sometimes on whether or not vasomotor symptoms cluster with other symptoms. Most previous studies in midlife women include symptoms defined a priori as being characteristic of menopause [9–17]. Cray and colleagues [9–11] conducted principal component and multilevel latent cluster analyses in the Seattle Women’s Midlife Health Study (SWMHS) using data from a 3-day symptom diary of 19 symptoms from six pre-defined symptom groups (hot flashes, sleep, pain, mood, cognitive and tension). One analysis suggested similar factor structures across the stages of reproductive aging [11]. However, another identified three latent classes: low symptom, low hot flash/moderate symptoms and high hot flash/moderate symptoms [10], that varied by menopausal stage. Women were less likely to be in either of the two latent classes that included hot flashes when they were premenopausal. In the MsFLASH clinical trial, latent class analysis based on hot flash, insomnia, sleep, depressed mood, anxiety and pain symptoms identified 5 classes, 4 of which included hot flashes [12]. Mishra and Dobson [13] conducted factor analysis to identify symptom clusters of 17 general health and menopausal symptoms using data from the Australian Longitudinal Study of Women’s Health (ALSWH). They identified four factors (somatic, urogynecological, vasomotor and physical). Longitudinal latent class analysis of each of the four symptom groups suggested that women clustered into patterns of mild, moderate, severe and very severe symptoms that remained consistent over time and across menopausal stages for all symptom groups except the vasomotor symptom group, which differed by the timing of change in severity scores.

Additional analyses of SWMHS suggest that symptom clustering was associated with both sex steroid hormones [9, 14] and cortisol levels [9]. A high symptom class was associated with decreased urinary cortisol levels while the high hot flash/aches/wakening class was associated with both higher urinary cortisol and lower urinary estrone levels [9]. A more recent analysis of symptom severity found that being in a class with severe hot flashes was associated with higher urinary follicle-stimulating hormone (FSH), lower urinary estrone and higher epinephrine levels but not with cortisol levels [14]. Greenblum and colleagues [15] using principal components analysis in a clinical sample identified three symptom clusters (psychological symptoms; weight gain and urinary incontinence; and, vaginal dryness and sleep disturbances), and reported that the vaginal dryness and sleep disturbance cluster was most strongly associated with self-reported quality of life.

Only a few studies have evaluated cross-cultural or race/ethnic differences in symptom profiles. The four country Decisions at Menopause Study (DAMES) found that hot flashes grouped with other symptoms differentially across countries [16]. Im and colleagues [17] reported race/ethnic differences in reporting of the number and severity of physical symptoms but only in the least symptomatic cluster. The MS-Flash study reported that Black women were more likely than White women to cluster in the severe hot flash, insomnia and pain cluster [12].

In the multiethnic Study of Women’s Health Across the Nation (SWAN), we have examined associations between pairs of symptoms [1–3] and a triad of symptoms (sleep disturbances, depressed mood and sexual problems) [18] while Avis and colleagues [19] considered evidence for a menopausal syndrome. In the present paper, we used longitudinal data from SWAN to conduct latent transition analysis to assess symptom clustering as women transition through the menopause. Unlike most prior studies, which defined symptom groups a priori, we used a more agnostic, data-driven approach utilizing information on all reported symptoms to construct latent classes of symptoms and estimate how women move between these classes over time. Like Mishra and Dobson [13], our aim is to understand the broader symptom experience of midlife women and whether symptom clustering differs by menopausal status. We further assess whether demographic characteristics, including race/ethnicity, body size or smoking status were associated with specific symptom clustering and evaluate the association between symptom clustering and women’s self-reported health status.

## Methods

This paper uses data from the longitudinal cohort study, the Study of Women’s Health Across the Nation, details of which have been described elsewhere [20]. In brief, eligible women were identified through a cross-sectional screening survey at seven clinical sites and enrolled in the cohort study. Eligibility for the cohort study included residence in the geographic area of the clinical site, being age 42–52 years old, self-identification as White (at all sites) or as Black (at the southeastern Michigan, Boston, Chicago or Pittsburgh sites), Chinese (at the Northern California site), Japanese (at the Southern California site) and Hispanic (at the New Jersey site), the ability to speak English, Cantonese, Japanese or Spanish and ability to give verbal consent. In addition, women had to have an intact uterus, at least one menstrual period and not have used reproductive hormones in the past 3 months, and could not be pregnant or lactating at the time of enrollment. The study protocol was approved by the Institutional Review Boards at each study site. A total of 3302 women were enrolled in 1996/1997 and followed approximately annually thereafter with 12 clinic visits completed by 2012, at which time the study remained in contact with over 80% of surviving participants. All participants provided written, informed consent at each visit.

Each visit included interviewer-administered and self-administered questionnaires on a broad range of topics, including menstrual characteristics, socio-demographic characteristics, lifestyle and physical, psychological, and menopausal symptoms. Physical assessments included measurement of height and weight.

As the set of questions asked varied by visit, we sought to maximize the number of questions related to women’s symptom profile while ensuring measurement consistency across multiple visits. Thus this analysis includes data derived from 58 questions included at the baseline visit, as well as follow-up visits 1, 2, 3, 6, 8, 10 and 12. A woman’s first observed visit in each stage of reproductive aging (premenopausal, early-perimenopause, late-perimenopause and post-menopause) was selected for inclusion in this analysis. Given timing of the visits, women were not always observed at all stages. Data from an individual visit were excluded when information on more than 10 of the included symptoms was missing, after a hysterectomy or bi-lateral oophorectomy, or when menopausal stage could not be classified because of HT use. Based on these exclusions 13 women had no eligible observations, leaving 3289 women eligible for this analysis.

### Menopausal stage

Menopausal stage was defined based on women’s self-reported menstrual characteristics at each visit. Women were classified as premenopausal if they had had a menstrual period in the previous 3 months and reported no change in menstrual regularity in the past 12 months; as early peri-menopausal if they reported decreased regularity in their menses in the past 12 months and had had a menstrual period in the previous 3 months; as late peri-menopausal if they had had no menses in the past 3–11 months; and, as postmenopausal if they had had no menses for the past 12 or more months. Surgical menopause was defined by report of either hysterectomy or bilateral oophorectomy. Women were censored at the time of surgical menopause (*n* = 237). At enrollment women were either pre- or early peri-menopausal. Of the 3289 women included in the analysis, 1761 were observed at least once in the premenopausal stage, 2777 in the early peri-menopausal stage, 927 in the late peri-menopausal stage, and 2222 in the early post-menopause.

### Symptoms

A total of 58 questions ascertained information on a broad range of symptoms. Although a number of these questions are items from existing scales intended to measure specific concepts (e.g., items from the CESD scale designed to measure depressive symptoms), this data-driven approach considers each item independently. This approach recognizes that women may differentially endorse specific questions in a scale and that examining the broader pattern of responses across large question sets may yield new insights. The questions included here were drawn from the SF-36 domains of role-physical, bodily pain, role-emotional, vitality and social functioning [21], the Center for Epidemiologic Studies Depression Scale (CES-D) [22, 23], the 4-item Cohen’s Perceived Stress Scale [24] as well as a 14-item list of general symptoms assessed in SWAN and other studies of the menopausal transition including vasomotor symptoms, mood symptoms, somatic symptoms and vaginal dryness [25–27]. For the latter, women were asked how often within the past 2 weeks (ranging from not at all to daily) they experienced each symptom. Additional questions included items related to self-reported sleep quality (trouble falling asleep and staying asleep, waking early and perceived sleep quality) [28, 29], as well as questions on involuntary urine loss [30] and sexual desire [31].

*Self-Reported Health* was assessed by the question “Would you say your health in general is excellent, very good, good, fair or poor?” [21].

### Covariates

Race/ethnicity was self-defined and categorized as Black, Chinese, Japanese, Hispanic or White. Information on highest level of education attained (high school graduate/GED or less than high school versus at least some college), economic strain and smoking status (current, past, never) was obtained at baseline. Economic strain was assessed with the question “how hard is it to pay for basics (very hard, somewhat hard or not hard)?” Height and weight were measured without shoes, and in light indoor clothing. BMI, calculated as weight in kilograms divided by height in meters squared, was categorized as underweight (<18.5 kg/m^2^), normal weight (18.5–24.9 kg/m^2^), overweight (25.0–29.9 kg/m^2^), or obese (≥30.0 kg/m^2^).

### Statistical analysis

Latent class analysis (LCA) [32] is a data reduction method for categorical variables akin to factor analysis for continuous variables. LCA estimates a probability *p*_*ijk*_ of response to the *kth* category of the *jth* symptom for the *ith* latent class. For example, based on our analyses, the appetite symptom “I did not feel like eating; my appetite was poor” is described by 44.4% of women in latent class 1 (most symptomatic) as occurring “rarely or none of the time” (=1), 26.6% as “some or a little of the time” (=2), 17.5% as “occasionally or a moderate amount of the time” (=3), and 11.6% as “most or all of the time” (=4). In contrast, this symptom is described by 84.5% of women in latent class 6 (minimally symptomatic) as occurring “rarely or none of the time”, 11.7% as “some or a little of the time”, 2.9% as “occasionally or a moderate amount of the time”, and 0.8% as “most or all of the time”.

By assuming women belong to unobserved (latent) groupings of reported symptoms, in which symptom reports are assumed to be independent conditional on latent class, a data-driven clustering of symptoms can be determined. As an initial exploratory step we conducted latent class analyses [32] cross-sectionally at each menopausal stage to assess whether latent classes differed across the menopausal transition. Analyses were conducted first with all women and then with only those women observed in all of the four menopausal stages to evaluate whether the latent class structure was sensitive to lost-to-follow up. These exploratory analyses indicated that latent classes remained consistent over time and across menopausal stage when a fixed set of symptoms were included (data not shown).

Latent Transition analysis (LTA) extends LCA to a longitudinal data setting [33, 34]. We used LTA to determine how symptoms cluster and to estimate how women transition between the identified clusters across menopausal stages (SAS PROC LTA) [33]. At each menopausal stage (pre-menopause, early peri-menopause, late peri-menopause and post-menopause) women are assumed to belong to one of *C* unobserved (“latent”) classes. Conditional on this latent class, each symptom is assumed to have a specific distribution, and again conditional on this latent class, all symptoms are assumed to be mutually independent. Along with clustering of symptoms into classes, LTA estimates a *C x C* transition matrix between latent classes for each time point. Based on the exploratory analysis above and to assist in interpretation, latent classes were kept constant across the time points.

The Bayesian Information Criterion (BIC) (which penalizes models with large numbers of latent classes to avoid overfitting), along with scientific judgement, was used to select the number of classes. Finally, since PROC LTA does not use multiple start points to ensure convergence of the LTA model, we introduced multiple start-points to ensure consistent estimation of the global maximum likelihood.

To summarize composition of latent classes (i.e., the distribution of symptomatology across the latent classes), we estimated average intensity of each symptom within each class. Since symptoms were measured on different scales (e.g. a few were dichotomous responses while others might have as many as six response levels), we standardized all symptom responses to a [0,1] scale, with 0 the most favorable category listed, and 1 being the worst. This symptom intensity for a given class was constructed as ${P}_{ij}={\displaystyle {\sum}_{k=1}^K{p}_{ij k}\left(\frac{k-1}{K-1}\right)}$, where *i* indexes the latent class, *j* the symptom question and *k* the category associated with the symptom question (where response options were consistently (re) ordered to run from “best” to “worst”). Thus 0 ≤ *P*_*ij*_ ≤ 1, with *low intensity* values corresponding to latent classes in which women rarely reported problems with that symptom and *high intensity* values (near 1) corresponding to latent classes in which women often reported problems with that symptom. For example, using the appetite symptom and response probabilities for latent classes 1 and 6 presented above, the intensity is *P*_1,*APPETITE*_ = 100 × (.444 × 0/3+.266 × 1/3+.175 × 2/3+.116 × 3/3) = 32.1 for latent class 1 and *P*_6,*APPETITE*_ = 100 × (.845 × 0/3+.117 × 1/3+.029 × 2/3+.008 × 3/3) = 6.6 for latent class 6. These intensity measures are then summarized into a “heat map” corresponding to the measures of *P*_*ij*_ to help interpret the symptom intensity distribution results of the latent classes produced by LTA. No a priori grouping of symptoms into specific domains was assumed. In order to enhance interpretation of the symptom distributions in each cluster, we ordered the symptoms in the heat map by intensity level across latent classes and grouped symptoms post hoc conceptually (e.g. sleep disturbance, pain, psychological).

Joint multinomial logistic regression was used to compute the odds of belonging to a given initial latent class relative to a reference initial latent class as a function of baseline covariates: age, obesity status, race/ethnicity, smoking status (current, past or never smoker), financial strain (very hard to pay for basics, hard to pay for basics, not hard to pay for basics), and education (high school graduate or less versus some college or more). A bootstrap was used to compute empirical confidence intervals for the final multivariable multinomial logistic regression on baseline latent classes.

In order to determine whether the latent classes were related to self-reported health (categorized as excellent, very good, good and fair or poor) at each time point, a four-level partial proportional odds regression model was fit using the estimated symptom latent class at each menopausal stage and menopausal status as predictors, adjusted for age, obesity status, race/ethnicity, smoking status, financial strain and education. This model was fit using SAS PROC NLMIXED with a subject-level random intercept to account for within-woman correlation across the four time points. Variation in uncertainty of the latent class assignment was addressed with entropy based design weights [34].

## Results

Women had a mean age of 45.7 years at baseline and a mean BMI of 27.2 kg/m^2^. The study population was 28.3% Black, 47.0% White, 7.6% Chinese, 8.5% Hispanic, and 8.5% Japanese (Table 1). One-quarter of the women had a high school education or less and about one-third reported they found it somewhat or very hard to pay for basics. Less than half had ever smoked. At baseline, the majority of women reported they had very good or excellent health, but 13.2% rated themselves as having fair or poor health.

**Table 1:** Baseline demographics of 3289 women in the analytic sample, Study of Women’s Health Across the Nation (SWAN)

	Total (*N* = 3289)	Per Cent
Study site		
Michigan	542	16.5%
Boston	448	13.6%
Chicago	456	13.9%
Davis	459	14.0%
Los Angeles	496	15.1%
New Jersey	425	12.9%
Pittsburgh	463	14.1%
Race/ethnicity		
Black	931	28.3%
White	1546	47.0%
Chinese	250	7.6%
Hispanic	281	8.5%
Japanese	281	8.5%
Education^a^		
Some college or more	2446	75.0%
High school or less	814	25.0%
Smoking status^b^		
Never	1867	57.3%
Past	824	25.3%
Current	567	17.4%
How hard it is to pay for basics^c^		
Very hard	302	9.2%
Somewhat hard	1002	30.7%
Not hard	1965	60.1%
Self-rated health^d^		
Excellent	695	21.4%
Very good	1179	36.3%
Good	945	29.1%
Fair	368	11.3%
Poor	62	1.9%

^a^29 Missing observations

^b^31 Missing observations

^c^20 Missing observations

^d^40 Missing observations

### Latent classes

**Fig. 1:**
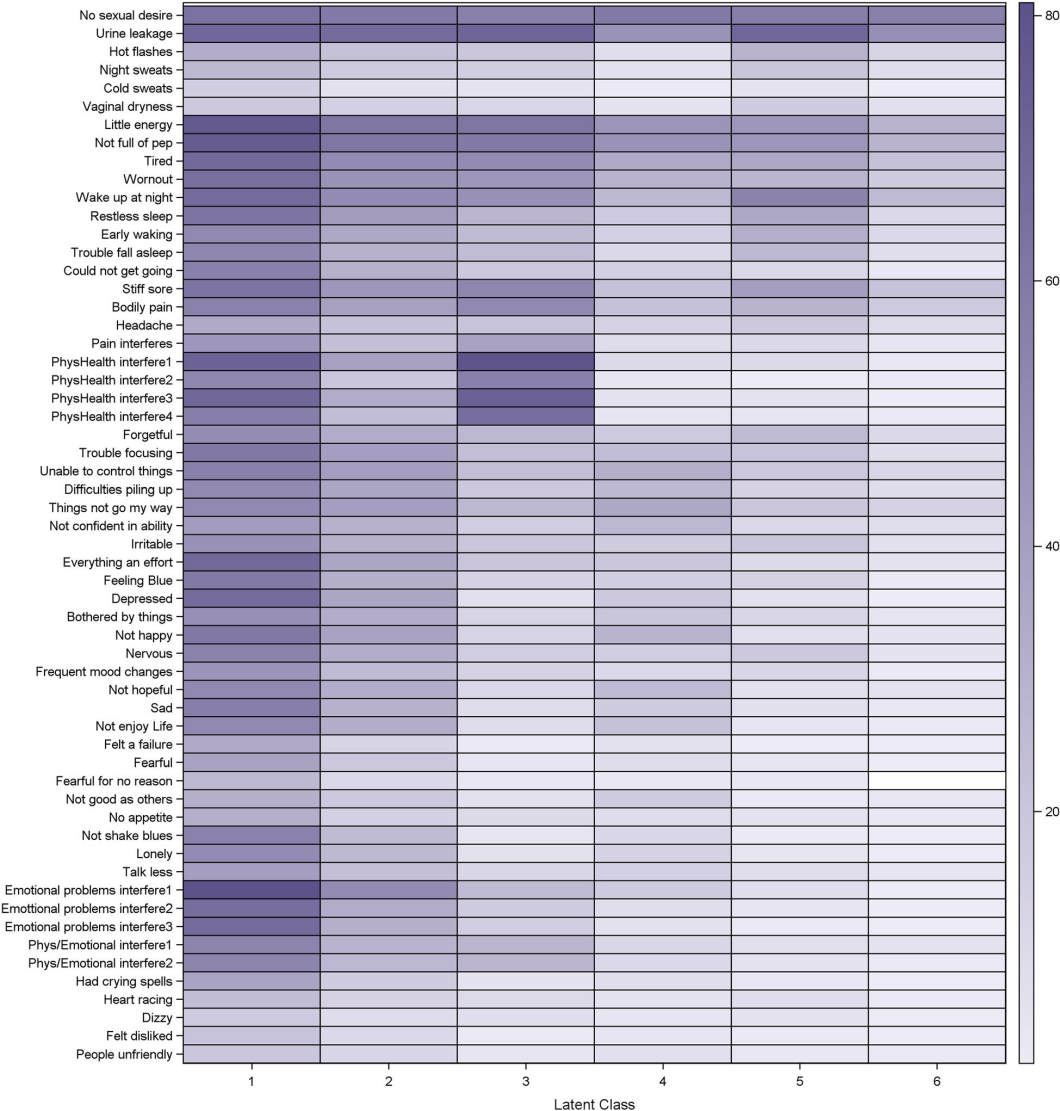
Heat map of symptom intensity by six defined latent classes with *darker blue* indicating higher intensity symptoms, Study of Women’s Health Across the Nation (SWAN)

The LTA identified 6 distinct latent classes. These classes were ordered based on the number and intensity of symptoms from 1 (most symptoms present and highest intensity) to 6 (least symptoms present and least intensity). Figure 1 provides the heat map showing symptom intensity of each symptom within each latent class. (Additional file 1 provides the full question item that corresponds with the shorter symptom labels provided in the heat map.) Latent Class 1 (LC1) is highly symptomatic with high intensity ratings for most measured symptoms. Latent Class 2 (LC2) is similar to LC1, but with moderate intensity rating for most measured symptoms. Latent Classes 3–5 each include fewer moderate to high intensity symptoms than LC1 or LC2. Latent Class 3 (LC3) includes moderate intensity vasomotor, pain, fatigue, sleep and physical health symptoms sufficient to interfere in life activities, but fewer and lower intensity psychological symptoms. Like LC1 and LC2, Latent Class 4 (LC4) includes numerous symptoms but of milder intensity, dominated by fatigue and psychological symptoms. Latent Class 5 (LC5) is similar to LC3 but does not include the physical health interference items. Latent Class 6 (LC6) is relatively asymptomatic with only a few, mild symptoms mostly related to fatigue. Notably, vasomotor symptoms tended to cluster with sleep disturbances and fatigue and were present in each of the moderate to highly symptomatic clusters (LC1,2,3 and 5). This triad represents the most intense symptoms only in LC5. Intensity of low sexual desire and urogenital symptoms differed little across classes.

### Probability of transition from latent class to latent class across reproductive stage

In the pre-menopause, fully 26% of women were classified as moderately to highly symptomatic: 10% in the highly symptomatic LC1 and 16% in the moderately symptomatic LC2. Another 40% were classified as moderately symptomatic for a subset of measured symptoms: 14% in LC3 and 26% in LC5. Another 14% were classified as mildly symptomatic (LC4) while 20% were classified in relatively asymptomatic cluster (LC6).

Because transition probabilities did not differ significantly across menopausal stages (*X* ^2^ = 76.84 on 64 *df*, *p*-value = 0.13), transition probabilities were set to be constant over time. Table 2 provides the probability of women in a given class transitioning to the same or a different class from one menopausal stage to the next. Although symptoms improved and/or worsened for some women, most women remained in their same class at each subsequent time-point.

**Table 2:** Latent class transition probabilities (latent classes numbered from maximal symptoms (1) to least symptoms (6)), Study of Women’s Health Across the Nation(﻿SWAN﻿)

		Probability of transition to latent class_*t+1*_				
Latent Class_t_	1	2	3	4	5	6
1	**0.55**	0.26	0.04	0.10	0.03	0.03
2	0.12	**0.47**	0.12	0.14	0.13	0.01
3	0.04	0.14	**0.39**	0.11	0.23	0.10
4	0.04	0.13	0.07	**0.44**	0.20	0.11
5	0.01	0.08	0.09	0.07	**0.62**	0.13
6	0.01	0.00	0.06	0.06	0.11	**0.76**

(the diagonal, in bol﻿d, is the proportion of women who remain in the same latent class)

**Fig. 2:**
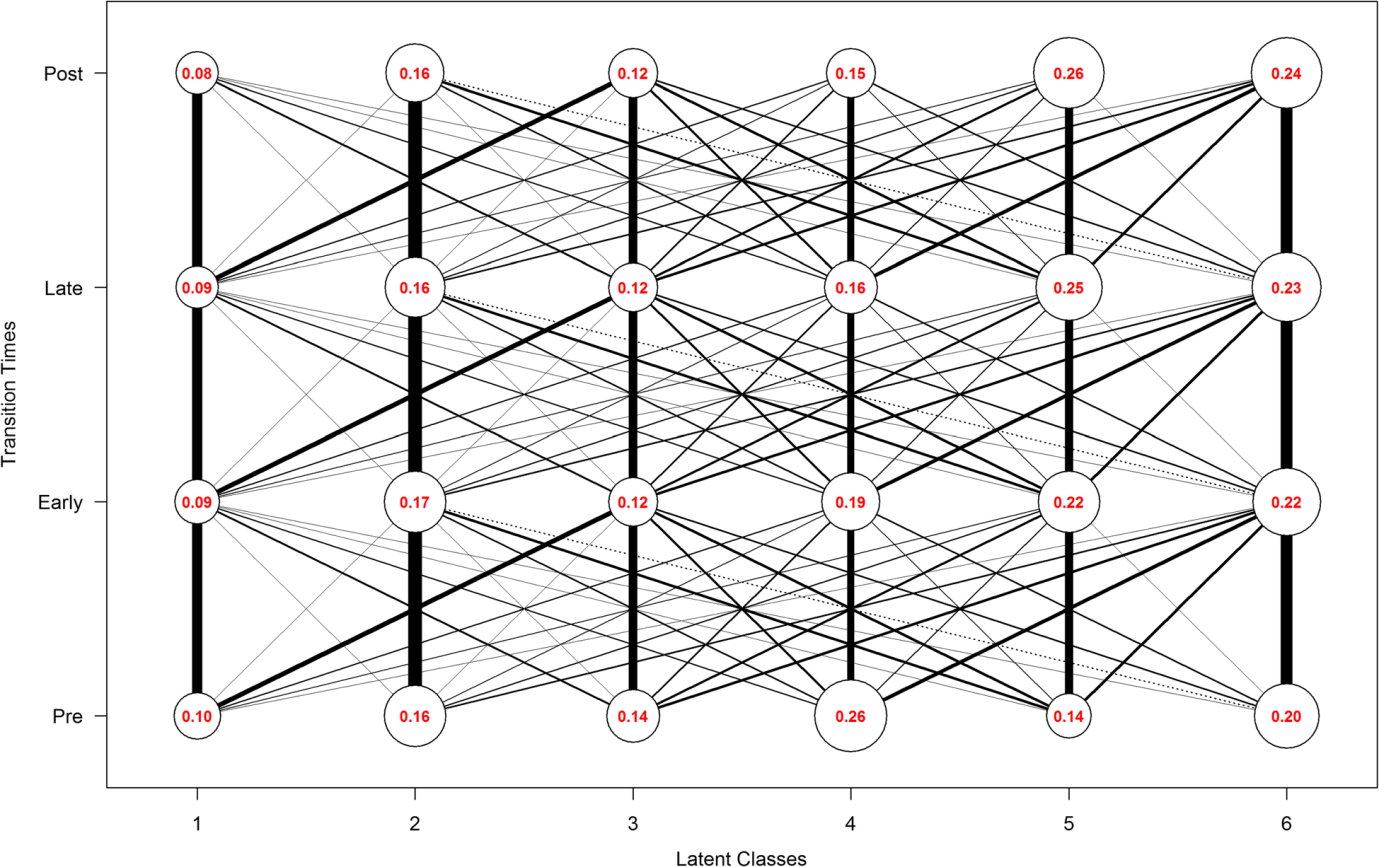
Proportion of women in each latent class (estimated probability presented in *circles* and *area of circle* proportional to the probability) by menopausal status and transition probabilities across menopausal stage (shown by *thickness and direction of lines*), Study of Women’s Health Across the Nation (SWAN)

Figure 2 illustrates how the LC transition probabilities affect the movement of women from one class to another and the resultant proportion of women in each latent class at each stage of the menopausal transition. The width of the lines represents the probability of moving from class to class. Thus the wide vertical lines illustrate that women were most likely to remain in their same class as they transitioned through menopause. The thinner diagonal lines represent the lower probability of movement between classes, particularly more distant classes. By post-menopause, the probability of being in each latent class was 8% for the most highly symptomatic LC1, 16%, 12%, 15% and 26% for latent classes 2–5, respectively and 24% for the least symptomatic LC6. The biggest differences by menopausal stage can be seen in LC4 and LC5: 26% of premenopausal women were in class 4 compared to 15% by the post-menopause whereas 14% of premenopausal women were in LC5 compared to 26% by the post-menopause.

### Characteristics associated with latent class membership

Table 3 presents the fully adjusted model for the association of baseline sociodemographic factors, obesity status and smoking status with the probability of being in each latent class compared to being in the least symptomatic LC6 (referent) at the premenopausal visit. After adjusting for all other covariates in the model, financial strain stands out as the variable most strongly and consistently related to symptoms. Having a somewhat or very hard time paying for basics was associated with over a five-fold and seventeen-fold increased odds, respectively, of being in the most symptomatic class (LC1) as well as with over a four-fold increased odds of being in the moderate symptom class (LC5) compared to women who did not report financial strain. Being obese was associated with a more than a two-fold odds of being in the highly symptomatic class (LC1) or the mildly symptomatic class (LC 5) : and with an 80% increase in the odds of being in the moderately symptomatic class (LC2) compared to non-obese women. Black women were one-half to one-third as likely to be in the more symptomatic clusters LC1 to LC3 than White women with similar characteristics, while Japanese were less likely to be in LC1 and Chinese women were less likely to be in LC2 than White women. Current smokers had a two and half fold increased odds and past smokers had a 75% increased odds of being in LC1 compared to never smokers. Older age at baseline was associated with slightly reduced odds of being in the more highly symptomatic LC1 and LC2, compared to younger women, although the confidence interval for the former includes 1.0. Level of education was not independently associated with latent symptom classes.

**Table 3:** Socio-demographic and lifestyle factors associated with premenopausal latent class ((latent classes numbered from most symptomatic (1) to less symptomatic (5), all compared to the least symptomatic (6, referent)), Study of Women’s Health Across the Nation (SWAN)

	Latent class (reference = LC6)				
	1	2	3	4	5
	OR (95% CI)	OR (95% CI)	OR (95% CI)	OR (95% CI)	OR (95% CI)
Age	**0.94 (0.89, 1.00)** ^a^	**0.92 (0.87, 0.98)**	1.04 (0.96, 1.13)	0.95 (0.88, 1.03)	1.00 (0.94, 1.06)
Body mass index					
Obese	**2.34 (1.46, 3.74)**	**1.80 (1.11, 2.94)**	1.25 (0.53, 2.95)	1.05 (0.47, 2.35)	**2.64 (1.11, 6.25)**
Not obese	ref	ref	ref	ref	ref
Race/ethnicity					
Black	**0.54 (0.30, 0.97)**	**0.42 (0.24, 0.76)**	**0.38 (0.16, 0.92)**	0.59 (0.31, 1.16)	0.59 (0.32, 1.11)
White	ref	ref	ref	ref	ref
Chinese	0.55 (0.18, 1.68)	**0.43 (0.19, 0.96)**	0.36 (0.10, 1.29)	1.20 (0.41, 3.52)	0.73 (0.34, 1.53)
Hispanic	1.16 (0.48, 2.81)	0.94 (0.37, 2.37)	0.26 (0.04, 1.82)	0.66 (0.26, 1.65)	1.55 (0.49, 4.94)
Japanese	**0.21 (0.06, 0.70)**	0.66 (0.28, 1.57)	0.37 (0.11, 12.80)	0.99 (0.32, 3.09)	0.58 (0.23, 1.42)
Smoking status					
Past	**1.75 (1.09, 2.80)**	1.13 (0.66, 1.91)	1.57 (0.81, 3.05)	1.08 (0.66, 1.77)	0.95 (0.55, 1.65)
Current	**2.53 (1.52, 4.22)**	1.42 (0.89, 2.27)	0.79 (0.38, 1.64)	0.95 (0.50, 1.82)	1.07 (0.61, 1.89)
Never	ref	ref	ref	ref	ref
How hard it is to pay for basics					
Very	**17.80 (8.50, 37.50)**	**4.85 (2.31, 10.20)**	1.46 (0.40, 5.33)	**3.63 (1.59, 8.27)**	1.72 (0.58, 5.04)
Somewhat	**5.75 (3.46, 9.58)**	**4.10 (2.66, 6.30)**	**2.01 (1.03, 3.92)**	**2.53 (1.38, 4.65)**	**2.01 (1.10, 3.70)**
Not at all	ref	ref	ref	ref	ref
Education					
HS or less	1.54 (0.94, 2.51)	1.04 (0.63, 1.73)	0.56 (0.23, 1.35)	1.05 (0.61, 1.82)	0.77 (0.42, 1.42)
Some college	ref	ref	ref	ref	ref

^a^Significant Odds Ratios are bolded

### Association between latent class membership and self-reported health status

**Fig. 3:**
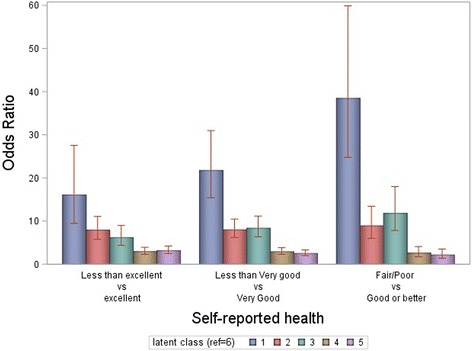
Adjusted Odds Ratios for different levels of self-reported health by latent class, Study of Women’s Health Across the Nation (SWAN)

Table 4 presents the regression coefficients for the association of self-reported health with menopausal stage and latent class membership adjusted for age, obesity, education, difficulty paying for basics, current smoking and race/ethnicity. (The distribution of self-reported health by menopausal status and latent class is provided in Additional file 2). Although, women in the late peri- and post- menopause were somewhat more likely to report being in fair to poor health compared to when they were premenopausal, women in the high to moderate symptomatic latent classes were much more likely to rate their health as fair to poor than women in the least symptomatic class. For example, based on the regression coefficients presented in Table 4, the odds of being in less than excellent health, less than very good health and less than good health were 3.56, 2.94 and 1.60 times higher for postmenopausal compared to premenopausal women, respectively. The comparable increased odds were 7.48, 8.21 and 9.73 times higher for women in LC1 compared to LC6. Figure 3 plots the odds ratios for each level of self-reported health by latent class to more clearly illustrate the magnitude of the association between LC and perceived health.

**Table 4:** Association of latent class and menopausal status with self-reported health^a^ (latent classes numbered from most symptomatic (1) to least symptomatic (6)), Study of Women’s Health Across the Nation

	Self-reported health Beta (95% CL)		
	Less than excellent vs. excellent	Less than very good vs. very good or better	Fair/poor vs. good or better
Menopausal status			
Pre	ref	ref	ref
Early peri	0.54 (0.32, .76)	0.34 (0.14,0.54)	0.12 (−0.15, 0.39)
Late peri	1.34 (1.01, 1.67)	0.89 (0.64, 1.14)	0.39 (0.04, 0.74)
Post	1.31 (1.07, 1.55)	1.08 (0.88, 1.28)	0.59 (0.32, 0.86)
Latent class			
1	2.75 (2.20,3.30)	3.02 (2.67, 3.37)	3.58 (3.13, 4.03)
2	1.99 (1.66, 2.32)	2.05 (1.78, 2.32)	2.14 (1.73, 2.55)
3	1.80 (1.43, 2.17)	2.09 (1.80, 2.38)	2.43 (2.00, 2.86)
4	1.06 (0.79, 1.33)	1.06 (0.81, 1.31)	0.91 (0.46, 1.36)
5	1.14 (1.39, 2.05)	0.92 (0.67, 1.17)	0.78 (0.31, 1.25)
6	ref	ref	ref

^a^Adjusted for obesity, education, difficulty paying for basics, current smoking, race/ethnicity and age

## Discussion

The evaluation of a broad range of physical and psychological symptom in a multi-ethnic cohort of midlife women contextualizes symptoms thought to be associated with menopause (e.g. hot flashes) within midlife women’s overall symptom experience. We identified six latent symptom classes that ranged from highly or moderately symptomatic across all measured symptoms, to moderately symptomatic for a subset of symptoms (vasomotor, pain, fatigue, sleep and physical health), to mildly symptomatic predominately associated with fatigue and psychological symptoms, to minimally or asymptomatic. Notably, vasomotor symptoms tended to cluster with sleep disturbances and fatigue, but were the most intense symptoms within only one mildly symptomatic latent class. Other symptoms often associated with menopause – low sexual desire and urogenital symptoms – were also not unique to, or of differential intensity, across the latent classes. This finding suggests that women may perceive these symptoms differently from other symptom domains, or that their underlying physiologic or social correlates differ from the other symptoms measured here. Although some women worsened or improved over time, they tended to track within latent class and menopausal stage did not influence the probability of movement between latent classes. Notably, one-quarter of the women were highly or moderately symptomatic in the pre-menopause and latent class was strongly associated with women’s self-perceived health.

These clustering patterns provide interesting insights into the aging and disablement processes. Sleep and fatigue symptoms, though mild, were present even in the least symptomatic cluster. As clusters became more symptomatic, sleep disturbance and fatigue symptoms worsened and pain symptoms emerged, becoming more prevalent and severe in the more symptomatic clusters. Psychological symptoms were prominent in just three of the six classes, but the two most highly symptomatic classes were characterized by having multiple, high/moderate intensity psychological symptoms.

The co-occurrence of sleep, fatigue and vasomotor symptoms has been reported by other studies [9] while SWAN has reported a triad of symptoms (sleep disturbances, depressed mood and sexual problems) associated with lower household incomes, less education and fair to poor self-rated health [18]. In the SWMHS, the high hot flash/aches/wakening cluster was associated with low estrone [9] while higher FSH and lower estradiol levels were associated with being in the severe hot flash cluster [14]. Future analyses of the SWAN data will assess the association between the identified symptom clusters and reproductive hormone levels.

Across previous studies, the number and content of factors/latent classes varies dependent on the number of women in the analytical sample, the number and types of symptoms considered, and the methodology used to identify clusters. For example, analyses from the SWMHS [9–11, 14] were based on a 3-day symptom diary that ascertained 47 symptoms, but the publications included fewer and a varied number of symptoms (i.e., 22, 19 or 15 symptoms or 5 indicator symptoms) and different subsets of the study population (*n* = 103 to 292 women). Nonetheless high/moderate symptom and low symptom profiles were evident in each analysis, with hot flashes often emerging more clearly in the low symptomatology clusters, as observed here.

The saliency of hot flashes in many studies may reflect their identification a priori as a study focus [10] or because the sample over-represented women who self-identified as having troublesome hot flashes [12]. The ALSWH included a broad range of symptoms in a population-based sample of women [13], identified vasomotor symptoms as one and uro-gynecological symptoms as another of four factors. However, the ALSWH study included a list of just 17 symptoms. In the present analysis, the triad of vasomotor symptoms, sleep disturbance and fatigue stood out uniquely only in an otherwise low symptomatic cluster (LC5), but were present in 5 of the 6 classes. Unlike the ALSWH, we found that intensity of low sexual desire and urogenital symptoms differed little across latent classes.

We found that the number and symptom content of the latent classes was consistent across menopausal stage. Similar symptom factor structures across stages of reproductive aging have also been reported in the SWMHS [11]. In the ALSWH, severity of women’s scores tended to remain stable over a 14-year period [13].

As might be expected, current and former smokers were more likely to be classified in the highly symptomatic cluster as were obese women. However, experiencing financial strain was the risk factor most strongly associated with being in the highly symptomatic clusters. Financial strain has been shown to be a major correlate of disability in SWAN [35] and other studies [36, 37]. Individuals with substantial financial limitations may be a particularly vulnerable group lacking health care access and related resources.

Only a few studies have evaluated cross-cultural or race/ethnic differences in symptom profiles. In the DAMES study [16], hot flashes did not cluster with other symptoms in the United States or Lebanon, but clustered with vaginal dryness and sexual symptoms in Spain and with general symptoms in Morocco. A multi-ethnic internet-based survey [17], observed differences by race/ethnicity only in the least symptomatic cluster: Black and White women reported more and greater severity of symptoms compared to Hispanic and Asian women. In contrast, the MS-Flash study reported that Black women were more likely than White women to cluster in the severe hot flash, insomnia and pain cluster [12]. In the present multi-ethnic study, after controlling for other covariates including financial strain, Black women but also Japanese and Chinese women were less likely to be classified in the most symptomatic latent classes.

Notably, one quarter of premenopausal women were highly or moderately symptomatic and experiencing numerous physical and psychological symptoms. Thus prior to beginning the menopausal transition a substantial proportion of women were highly symptomatic. Consistent with other studies examining the impact of high symptomatology on quality of life [7, 10], women in the high symptomatic cluster were most likely to perceive themselves in fair to poor health. The SWAN study population excluded women who experienced menopause earlier than their same age peers and women who were surgically menopausal. Given that earlier menopause and surgical menopause is associated with poorer health and mortality [38–41], participants were likely healthier than the general population of midlife women. Thus, this analysis may underestimate the proportion of women who are highly symptomatic as they enter the midlife, as further evidenced by the fact that older women at baseline tended to be in the less symptomatic latent classes. Further evaluation of symptom burden in the midlife may provide increased understanding of risk factors for the development of multiple morbidities and disability in later life. In the ALSWH, among women aged 76–81 years, multi-morbidity in the musculoskeletal/somatic, neurological/mental health and cardiovascular domains were each associated with poorer function as measured by Activities of Daily Living and the Instrumental Activities of Daily Living Scales [42].

This study has some limitations. As noted above, the SWAN cohort was left truncated and likely excluded women in poorer health [38]. Similarly, women who were lost to follow-up may have been in poorer health. Thus the burden of high symptomatology in midlife women may be underestimated. Although some bias may exist, retention in the SWAN cohort is fairly high (81% of the living participants at visit 12). Results from the latent class analyses were similar when we used complete cases and all cases, and the findings were robust across visits and menopausal status. Finally, assessment of urogenital symptoms as well as sexual desire was limited to only one question each and may not have fully ascertained women’s symptom experience in these domains.

The study has several strengths. This analysis includes data from a community based and multiethnic cohort of over 2900 midlife women followed longitudinally, enabling us to evaluate the stability of latent classes and transition probabilities over time and across the stages of the menopausal transition in a large sample of women and to evaluate potential race/ethnic differences. As identification of latent class structure is dependent on the symptom domains included in the analysis and, to a lesser extent, on the number of symptoms allocated to a given domain, our inclusion of 58 symptom responses from a broad range of symptom domains, without a priori selection to highlight purported menopausal symptoms, enabled us to characterize women’s menopausal experience within their broader life and health experience.

## Conclusions

This paper illustrates that midlife women experience substantially different symptom burdens, that a large fraction of women report a significant symptom burden prior to the onset of the menopausal transition, that high and intense symptomatology across physical and psychological domains is strongly associated with financial strain, and that a high symptom burden influences women’s perception of being in fair or poor health. Vasomotor symptoms cluster with sleep disturbances and fatigue, but are of unique salience to women’s symptom burden only in a relatively small subset of women. Given that one-quarter of midlife women were already highly or moderately symptomatic in the pre-menopause, ensuring access to chronic disease preventive strategies in the early midlife is likely critical to ameliorating risk in the most vulnerable populations. Development of rigorous, evidence-based protocols for health and functional evaluations, inclusive of physical and mental health assessments as well as prevention and intervention guidance for women as they reach the midlife is likely warranted. Future studies should evaluate whether women with high symptomatology as they enter the midlife are at risk of premature mortality or earlier onset of disability, and whether low symptomatology at the onset of this life stage is a marker of healthy aging.

## Additional files

Additional file 1: Questions included in this analysis of symptom clusters and the corresponding labels used in the heat map. (PDF 213 kb)

Additional file 2: Distribution of self-reported health by latent class and menopausal status, study of women’s health across the nation (SWAN). (PNG 120 kb)

Additional file 3: Institutional Review Board approval information for each study site.ᅟ(DOCX 12 kb)
