# Supplementary material for: It is not just menopause: symptom clustering in the Study of Women’s Health Across the Nation
Source: Womens Midlife Health. 2017 Jul 27;3:2. doi: 10.1186/s40695-017-0021-y (PMC5760187; doi:10.1186/s40695-017-0021-y)
Supplement: Supplementary file 1 — Questions included in this analysis of symptom clusters and the corresponding labels used in the heat map. (PDF 213 kb) [file 40695_2017_21_MOESM1_ESM.pdf]

Additional file 1: Heatmap labels, question and source of question

LABEL	QUESTION	SCALE
NoSexualDesire	During the past 6 months, how often have you felt a desire to engage in any form of sexual activity, either alone or with a partner?	Sexual Desire
UrineLeakage	Since your last study visit, have you ever leaked, even a very small amount, of urine involuntarily?	Symptom Lists
HotFlashes	Over the past two weeks, how often have you had hot flashes or flushes?	Symptom Lists
NightSweats	Over the past two weeks, how often have you had night sweats?	Symptom Lists
ColdSweats	Over the past two weeks, how often have you had cold sweats?	Symptom Lists
VaginalDryness	Over the past two weeks, how often have you had vaginal dryness?	Symptom Lists
LittleEnergy	During the past 4 weeks, how much time did you have a lot of energy?	SF-36
NotFullofPep	How much of the time during the past 4 weeks did you feel "full of pep"?	SF-36
Tired	During the past 4 weeks, how much time did you feel tired?	SF-36
Wornout	During the past 4 weeks, how much time did you feel worn out?	SF-36
WakeUpat Night	In the past two weeks, how often did you wake up several times at night?	Symptom Lists
RestlessSleep	My sleep was restless	CESD
EarlyWaking	In the past two weeks, how often did you wake up earlier than you had planned to, and were unable to fall asleep again?	Symptom Lists
TroubleFallASleep	In the past two weeks, how often did you have trouble falling asleep?	Symptom Lists
CouldNotGetGoing	I could not get going	CESD
StiffSore	Over the past two weeks, how often have you had stiffness or soreness in joints, neck or shoulder?	Symptom Lists
BodilyPain	How much bodily pain have you had during the past 4 weeks?	SF-36
Headache	Over the past two weeks, how often have you had headaches?	Symptom Lists
PainInterferes	During the past 4 weeks, how much did pain interfere with your normal work (including both work outside the home and housework)?	SF-36
PhysHealthInterfere1	During the past 4 weeks, have you had any of the following problems with your work or other regular daily activities as a result of your physical health--(item b) accomplished less than you would like?	SF-36
PhysHealthInterfere2	During the past 4 weeks, have you had any of the following problems with your work or other regular daily activities as a result of your physical health--(item a) Cut down the amount of time you spent on work or other activities?	SF-36
PhysHealthInterfere3	During the past 4 weeks, have you had any of the following problems with your work or other regular daily activities as a result of your physical health--(item d) Had difficulty performing the work or other activities (for example, it took extra effort)?	SF-36
PhysHealthInterfere4	During the past 4 weeks, have you had any of the following problems with your work or other regular daily activities as a result of your physical health--(item c) Were limited in the kind of work or other activities?	SF-36
Forgetful	Over the past two weeks, how often have you had forgetfullness?	Symptom Lists
TroubleFocusing	I had trouble keeping my mind on what I was doing	CESD
UnableToControlThings	Felt unable to control important things in your life?	Cohen's Perceived Stress
DifficultiesPilingUp	Felt difficulties were piling so high that you could not control them?	Cohen's Perceived Stress
ThingsNotGoMyWay	Felt that things were going your way	Cohen's Perceived Stress
NotConfidantInAbility	Felt confident about your ability to handle personal problems	Cohen's Perceived Stress
Irritable	Over the past two weeks, how often have you had irritability or grouchiness?	Symptom Lists
EverythingAnEffort	I felt that everything I did was an effort	CESD
FeelingBlue	Over the past two weeks, how often have you had feeling blue or depressed?	Symptom Lists
Depressed	I felt depressed	CESD
BotheredByThings	I was bothered by things that usually don't bother me.	CESD
NotHappy	I was happy	CESD
Nervous	Over the past two weeks, how often have you had feeling tense or nervous?	Symptom Lists
FrequentMoodChanges	Over the past two weeks, how often have you had frequent mood changes?	Symptom Lists
NotHopeful	I felt hopeful about the future	CESD
Sad	I felt sad	CESD
NotEnjoyLife	I enjoyed life	CESD
FeltAFailure	I thought my life had been a failure	CESD
Fearful	I felt fearful	CESD
FearfulForNoReason	Over the past two weeks, how often have you had feeling fearful for no reason?	Symptom Lists
NotGoodAsOthers	I felt that I was just as good as other people	CESD
No Appetite	I did not feel like eating; my appetite was poor	CESD
Not Shake Blues	I felt that I could not shake off the blues even with help from my friends.	CESD
Lonely	I felt lonely	CESD
Talkless	I talked less than usual	CESD
EmotProblemsInterfere1	During the past 4 weeks, have you had any of the following problems with your work or other regular daily activities as a result of any emotional problems (such as feeling depressed or anxious)---(item b) Accomplished less than you would like?	SF-36
EmotProblemsInterefere2	During the past 4 weeks, have you had any of the following problems with your work or other regular daily activities as a result of any emotional problems (such as feeling depressed or anxious)---(item c) Didn't do work or other activities as carefully as usual?	SF-36
EmotProblemsInterefere3	During the past 4 weeks, have you had any of the following problems with your work or other regular daily activities as a result of any emotional problems (such as feeling depressed or anxious)---(item a) Cut down the amount of time you spent on work or other activities?	SF-36
PhyEmotInterfere1	During the past 4 weeks, how much of the time has your physical health or emotional problems interfered with your social activities (like visiting with friends, relatives, etc.)?	SF-36
PhyEmotInterfere2	During the past 4 weeks, to what extent has your physical health or emotional problems interfered with your normal social activities with family, friends, neighbors, or groups?	SF-36
HadCryingSpells	I had crying spells	CESD
HeartRacing	Over the past two weeks, how often have you had heart pounding or racing?	Symptom Lists
Dizzy	Over the past two weeks, how often have you had dizzy spells?	Symptom Lists
FeltDisliked	I felt that people disliked me	CESD
PeopleUnfriendly	People were unfriendly	CESD
