# Supplementary figures and images for: It is not just menopause: symptom clustering in the Study of Women’s Health Across the Nation
Source: Womens Midlife Health. 2017 Jul 27;3:2. doi: 10.1186/s40695-017-0021-y (PMC5760187; doi:10.1186/s40695-017-0021-y)

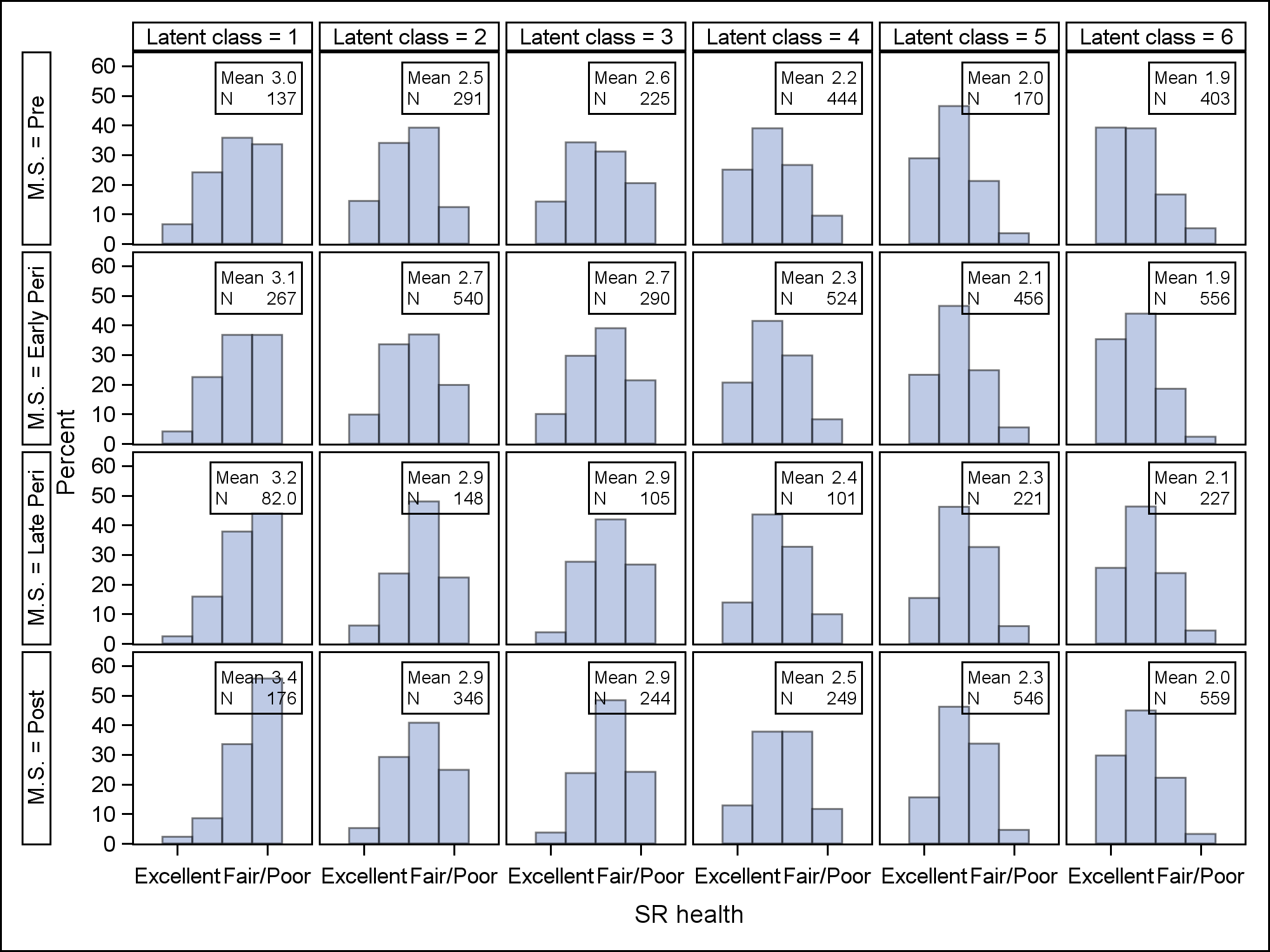

Supplement: Supplementary file 2 — Distribution of self-reported health by latent class and menopausal status, study of women’s health across the nation (SWAN). (PNG 120 kb) [file 40695_2017_21_MOESM2_ESM.png]
