# Supplementary material for: It is not just menopause: symptom clustering in the Study of Women’s Health Across the Nation
Source: Womens Midlife Health. 2017 Jul 27;3:2. doi: 10.1186/s40695-017-0021-y (PMC5760187; doi:10.1186/s40695-017-0021-y)
Supplement: Supplementary file 3 — Institutional Review Board approval information for each study site.ᅟ(DOCX 12 kb) [file 40695_2017_21_MOESM3_ESM.docx]

| **SWAN SITE** | **FWA#** | **IRB** |
| --- | --- | --- |
| Coordinating Center | **FWA00006790** (University of Pittsburgh), **FWA00006735** (University of Pittsburgh Medical Center), **FWA00000600** (Children’s Hospital of Pittsburgh), **FWA00003567** (Magee-Women’s Health Corporation), **FWA00003338** (University of Pittsburgh Medical Center Cancer Institute) | University of Pittsburgh  **IRB#: REN15070236/IRB0709006** |
| MGH | **FWA00003136** | Partners Healthcare  **IRB#: 1999P006353/MGH** |
| Michigan | **FWA00004969** | University of Michigan  **IRB#: 00000245** |
| New Jersey | **FWA0000014D** (Yeshiva University)  **FWA00002558** (Montefiore Medical Center)  **FWA00009807** (North Bronx Healthcare Network | Albert Einstein American College of Medicine of Yeshiva University  **IRB#: 2005-012** |
| Rush University Medical Center | **FWA00000482** | Rush University Medical Center  **IRB#: 13021201-IRB01-AM04** |
| UC Davis | **FWA00004557** | University of California, Davis **IRB# 260339-17** |
| UCLA | **FWA00004642** | UCLA Office of the Human Research Protection Program **IRB#11-002274-AM-00009** |
| University of Pittsburgh | **FWA00006790** (University of Pittsburgh), **FWA00006735** (University of Pittsburgh Medical Center), **FWA00000600** (Children’s Hospital of Pittsburgh), **FWA00003567** (Magee-Women’s Health Corporation), **FWA00003338** (University of Pittsburgh Medical Center Cancer Institute). | University of Pittsburgh  **REN16020248/IRB0402168** |
